# Supplementary material for: Mechanisms governing the pioneering and redistribution capabilities of the non-classical pioneer PU.1
Source: Nat Commun. 2020 Jan 21;11:402. doi: 10.1038/s41467-019-13960-2 (PMC6972792; doi:10.1038/s41467-019-13960-2)
Supplement: Supplementary file 7 — Source data [file 41467_2019_13960_MOESM7_ESM.zip › Source_Data/FigureS2/FigureS2B_MotifScanOutput/KmeansCluster2/homerResults/motif40.similar.html]

motif40

## Information for motif40

A
C
G
T
G
T
A
C
A
G
T
C
A
G
C
T
A
C
T
G
C
G
T
A
T
C
A
G
C
T
A
G
A
G
T
C
A
T
G
C
A
C
G
T
  
Reverse Opposite:  

C
G
T
A
A
T
C
G
C
T
A
G
G
A
T
C
A
G
T
C
C
G
A
T
A
G
T
C
C
T
G
A
C
T
A
G
A
C
T
G
C
G
T
A
  

|  |  |
| --- | --- |
| p-value: | 1e-27 |
| log p-value: | -6.233e+01 |
| Information Content per bp: | 1.921 |
| Number of Target Sequences with motif | 94.0 |
| Percentage of Target Sequences with motif | 1.87% |
| Number of Background Sequences with motif | 159.8 |
| Percentage of Background Sequences with motif | 0.39% |
| Average Position of motif in Targets | 91.0 +/- 80.3bp |
| Average Position of motif in Background | 98.4 +/- 71.6bp |
| Strand Bias (log2 ratio + to - strand density) | -0.4 |
| Multiplicity (# of sites on avg that occur together) | 1.00 |
| Motif File: | file (matrix) reverse opposite |

### Similar de novo motifs found

|  |  |  |  |  |  |  |  |
| --- | --- | --- | --- | --- | --- | --- | --- |
| Rank | Match Score | Redundant Motif | P-value | log P-value | % of Targets | % of Background | Motif file |
| 1 | 0.790 | C T A G T C A G C T A G C G T A A T C G C T A G A T G C T A G C A C G T A G T C T G C A C T A G | 1e-22 | -51.764876 | 1.79% | 0.44% | motif file (matrix) |
| 2 | 0.761 | C A T G C T A G C T A G C T A G C G T A A T C G C T A G G T A C A T G C A C G T A G T C T G C A A C T G | 1e-17 | -40.604572 | 1.19% | 0.25% | motif file (matrix) |
